# Pink Cricket Balls Through Rose-Tinted Glasses: Enhancing Interceptive Timing

**DOI:** 10.1177/2041669517743991

**Published:** 2017-11-29

**Authors:** Joshua M. Adie, Derek H. Arnold

**Affiliations:** School of Psychology, The 1974 University of Queensland, Brisbane, Australia

**Keywords:** motion perception, interceptive timing, luminance contrast

## Abstract

Cricket is a popular but potentially dangerous sport. It is played with a hard ball that can travel at great speeds. Serious injuries, including fatalities, have occurred when balls have struck participants. The game is traditionally played during daylight with a dark red ball, but recent games have been played during the day *and* at night using a ‘pink’ ball. We have reported data that seemed to justify concerns raised regarding the visibility of these new pink balls, as they were revealed to have a very low luminance contrast against pertinent backgrounds during twilight. Here, we report on the findings of a psychophysical experiment, wherein we mimicked twilight lighting conditions in an interceptive timing experiment using a pink moving disc as an analogue for pink cricket balls. We show that interceptive timing performance is diminished in conditions that mimic twilight. More importantly, we show that wearing glasses with a rose-tinted filter can alleviate this adverse impact by enhancing the luminance contrast of the pink ‘ball’ relative to pertinent backgrounds.

Cricket is one of the world’s oldest sports. It is played outdoors, traditionally in daylight hours with a dark red ball while players wear white clothing. This ensures that the ball is highly visible, as it has a high luminance contrast against pertinent backgrounds (see Adie & Arnold, 2017). More recently, cricket has been played during the day and at night (under lights) using a lighter ‘pink’ cricket ball. This colour selection was presumably motivated by a desire to enhance ball contrast against the dark night sky. Anecdotally, however, participants have complained about the visibility of these balls during twilight, and we have reported light readings taken during a match that seemed to confirm these complaints (see Adie & Arnold, 2017).

Issues regarding the visibility of pink cricket balls at twilight are related to changes in the intensity and composition of light during play. During traditional playing hours, in optimal viewing conditions, the sun can be said to appear yellow due to the dominant wavelength of light it emits (Appleton, 1945). As sunset approaches, light passes through an increasing volume of atmospheric particles before reaching our eyes. This disproportionately scatters higher frequency wavelengths of light, resulting in the sky seeming to take on a reddish hue due to changes in light composition (McCartney, 1976). Once stadium lights become dominant, the composition of lighting changes once more, back toward a more central wavelength within the visible spectrum.

We found that changes in the composition of light altered the luminance contrast of pink cricket balls relative to pertinent backgrounds during play. Bear in mind that the intensity of light reflected from a surface that looks red (or pink) will tend to become proportionally greater at sunset than at midday, relative to a surface that looks yellow (like the pitch – a hardened area of grass in the centre of the playing area) or green (like the outfield – grassed regions surrounding the pitch). Our light readings showed that the pink cricket ball was darker than the pitch, outfield, and the sky during normal daylight playing hours, but it lightened relative to all these surfaces during twilight, becoming equally bright with the sky about sunset. Why might this matter?

Human motion perception is primarily driven by encoded differences in brightness. It is therefore possible for a moving object to be ‘visible’ because it has a distinctive colour relative to its background, without people being able to accurately judge its speed, as it is nearly equally bright relative to the background (Cavanagh, Tyler, & Favreau, 1984; Lu, Lesmes, & Sperling, 1999). In extreme laboratory conditions, a phenomenon known as motion standstill can be induced, wherein a stimulus is clearly seen but its movement cannot be discerned at all – it seems to ‘standstill’ (see Arnold, Petrie, & Johnston, 2017; Lu et al., 1999). Changes in motion perception with lighting in real-world settings are unlikely ever to be as dramatic. They will, however, likely impact on the accuracy of interceptive timing tasks (like catching or hitting a cricket ball) as these rely on the precision of motion computations, which is relatively poor at low luminance contrasts (see Stone & Thompson, 1992).

To investigate what impact changes in lighting during day/night cricket matches might have on interceptive timing, we conducted an experiment that mimicked lighting conditions at twilight (∼1 hour before sunset) and at sunset on the day that we took light readings at a first-class cricket match in Brisbane, Australia (see Adie & Arnold, 2017). We constructed a stimulus that approximated the visual conditions from the perspective of a batsman, with a simulated sightscreen (in this form of cricket, a large white screen at either end of the ground), outfield, pitch, and a moving pink ball (see Figure 1). Luminance values were scaled and set to levels that matched luminance contrasts between the pink ball and the sightscreen, the outfield and the pitch at twilight and at sunset. As the pink ball had been nearly equally bright relative to the pitch and outfield at twilight, but had been brighter than these surfaces at sunset, we predicted that interceptive timing performance would be worse at ‘twilight’. We also explored a manipulation that might correct these adverse conditions – we had people wear rose-tinted sunglasses, which darken all colours, but relatively lighten the ball relative to different coloured (brown and green) surfaces.

**Figure 1.**
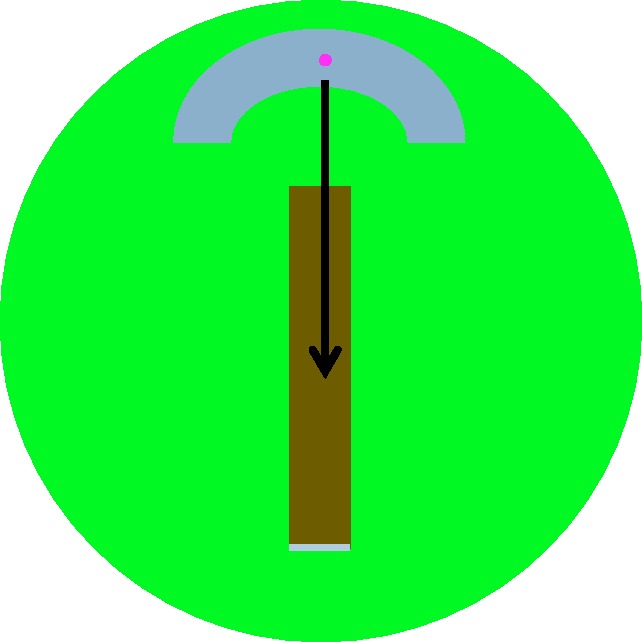
Graphic depicting the stimulus.

## Method

### Participants

Thirty participants (21 women, *M*_age_ = 20 years, *SD* = 5) volunteered to participate. All were undergraduate psychology students who were naïve as to the purpose of the experiment and were awarded course credit for participation. All participants reported having normal colour vision and normal, or corrected-to-normal, visual acuity. We note that many people with anomalous colour vision are unaware of their deficit, but do not believe we tested any as none of our participants experienced extreme disproportionate difficulty in low luminance contrast conditions. All participants provided informed consent before participating, and were advised that they could withdraw from the study at any time without penalty. This experiment was granted ethical approval from the University of Queensland Ethics Committee and was carried out in accordance with the Code of Ethics of the World Medical Association (Declaration of Helsinki).

### Apparatus

Stimuli were presented on a 20′′ HP P1110 monitor and generated by a Cambridge Research Systems ViSaGe stimulus generator driven by custom Matlab R2007b (The MathWorks, Natick, MA) software. The monitor had a resolution of 1024 × 768 pixels and a refresh rate of 85 Hz. The monitor was colour calibrated using a ColorCAL MKII Colorimeter, interfaced with the ViSaGe desktop monitor calibration routines. Participants viewed stimuli binocularly in a quiet darkened room from a distance of 57 cm, with their head positioned on a chin rest.

### Stimuli and Design

The stimulus comprised a pink disc (CIE 1931: x = 0.53, y = 0.29 Y = 12), a green circle (CIE 1931: x = 0.29, y = 0.42), a white/grey arc and line (CIE 1931: x = 0.29, y = 0.35) and a brown rectangle (CIE 1931: x = 0.35, y = 0.38). All chromatic coordinates were set to match readings from surfaces taken at a first-class cricket match (see Adie & Arnold, 2017), mimicking the pink ball, the outfield, the sightscreen and the pitch. The diameter of the ‘outfield’ subtended 25° of visual angle at the retina (dva). The ‘sightscreen’ arc was superimposed on the outfield. It had an angular subtense of 45°, with an outer and inner edged 12 and 11 dva from the centre of the field, respectively (see Figure 1). The ‘pitch’ was horizontally centred on the field and vertically centred 2.4 dva below the field centre. It had a width subtending 2.4 dva and a length subtending 14.4 dva. The ‘crease’ was superimposed on the lower edge of the pitch, with a width subtending 2.4 dva and a height of 0.25 dva. The ‘ball’ had a diameter subtending 0.25dva.

At the start of each test presentation, the ball was static, centred on the sightscreen (see Figure 1). This configuration was visible for 1.25 s, before a warning flashed black screen was shown (for 0.2 s), then the static configuration was represented for 0.5 s before the onset of ball motion. The ball then translated vertically down the display at a speed of 14.4, 15.8, 17.3, 19.3, 20.7. 22.4, or 24.4 dva/s. The ball stopped when it reached the edge of the outfield. Participants attempted to press a mouse button as the ball crossed the crease. Feedback was provided 1.5 s after ball motion onsets, with the ball re-positioned onto the point it had occupied when the participant pressed the mouse button for 1 s. Two tones were then sounded before the start of the next trial.

There were two simulated lighting conditions – twilight and sunset. In the twilight condition, the sightscreen (and crease) had a luminance intensity of 15 cd/m^2^ and in the sunset condition it had an intensity of 13.2 cd/m^2^, creating Michelson luminance contrasts of 11 and 5%, respectively. In the twilight condition, the pitch and field had luminance intensities of 12 cd/m^2^ and in the sunset condition they had intensities of 9 cd/m^2^, resulting in these surfaces being physically equiluminant with the ball in the twilight condition, and in a 14% Michelson contrast relative to the ball in the sunset condition. Luminance settings were set to mimic luminance contrasts between the pink ball and other surfaces during a first-class cricket match (see Adie & Arnold, 2017). While not reported in that paper, luminance readings from the pitch were closely matched to those taken from the outfield, so in these experiments physical green and brown luminance settings were equated. We focused our experiments on mimicking twilight and sunset luminance contrast conditions, as we wanted to know if the small physical luminance contrast differences brought about by changes in the composition of lighting around sunset would have a discernable impact on interceptive timing.

During a block of trials, each test speed was presented 20 times for each condition – all in random order, resulting in 280 individual trials. Timing errors, between participant button presses and times at which balls crossed the crease, were recorded on each trial. Participants completed two blocks of trials. In one block, they viewed the display without glasses. In the other, they wore sunglasses with rose-tinted lenses (Oakley Prizm Golf). These lenses darkened all test colours, but had less impact on pink. Pink luminance intensity was changed from 12 to 6.6 cd/m^2^, white/grey from 15 (Twilight) and 13 (Sunset) to 4.3 and 3.7 cd/m^2^, while the field and pitch were changed from 12 (Twilight) and 9 (Sunset) to 3.8 and 2.9 cd/m^2^, respectively. Block order was counterbalanced across participants to control for practise effects.

## Results

**Figure 2.**
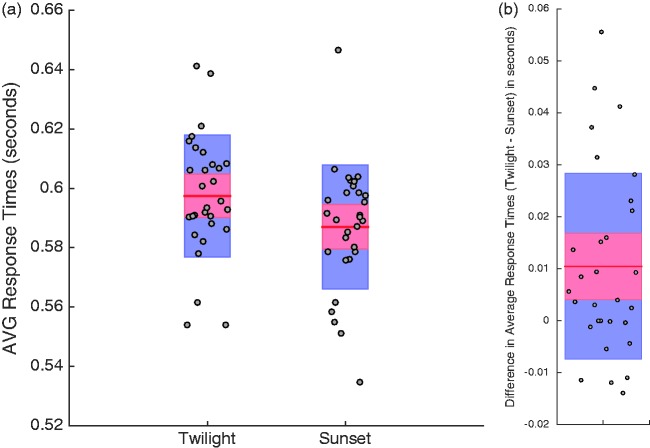
(a) Average participant response times to test presentations in the twilight and sunset conditions. (b) Differences between individual response times to test presentations in the twilight and sunset conditions. In both plots, individual data points are depicted by circular points, 95% CI limits by red shaded regions, and ± 1 *SD* by blue shaded regions. All data are from the block of trials completed while NOT wearing glasses.

While not wearing glasses, participants were (on average) slightly slower to respond on twilight (*M = *.6 *SD* = 0.02; see Figure 2(a)) relative to sunset (*M = *0.59 *SD* = 0.02; see Figure 2(a)) trials, *t*(29) = 3.2, *p* = .003, 95% CIs [0.004, 0.017]; see Figure 2(b). This is consistent with perceived speeds tending to be *slowed* at low luminance contrasts (Stone & Thompson, 1992; but also see Thompson, Brooks, & Hammett, 2006). The slight magnitude of this effect (∼10 ms) suggests to us, however, that people were able to somewhat calibrate average response times due to the provision of feedback.

**Figure 3.**
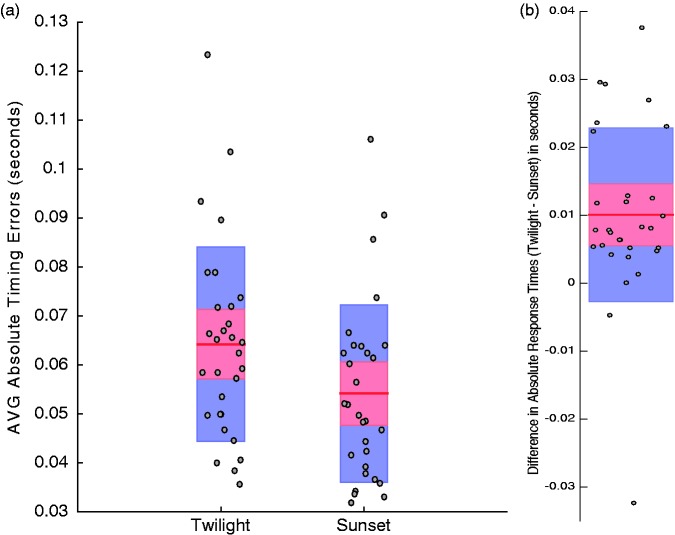
(a) Average absolute timing errors for participants on trials in the twilight and sunset conditions. (b) Differences between absolute timing errors for individuals during twilight and sunset trials. In both plots, individual data points are depicted by circular points, 95% CI limits by red shaded regions, and ± 1 *SD* by blue shaded regions. Individual data points in each plot are randomly jittered along the X axis to avoid superimposition. All data are from the block of trials completed while NOT wearing glasses.

We are more interested in *absolute* timing errors, as in an interceptive task (like hitting or catching a cricket ball) it does not matter if you are early or late – both result in missing or catching errors. We therefore calculated absolute (unsigned) timing errors for each trial (differences between button press times and when the moving ball was centred on the crease). Note that this measure of trial-by-trial precision can be quantified even when people are, on average, accurate in terms of their timing. Early and late trials that can average to a 0 timing offset will nonetheless contribute to an estimate of absolute trial-by-trial timing errors. We found that these were greater on twilight (*M* = 64 *SD* = 20 ms; see Figure 3(a)) than on sunset (*M = *54 *SD = *18 ms; see Figure 3(a)) trials (*t*_29_ = 4.3, *p* = .0002, 95% CIs [0.005, 0.015]; see Figure 3(b)).

**Figure 4.**
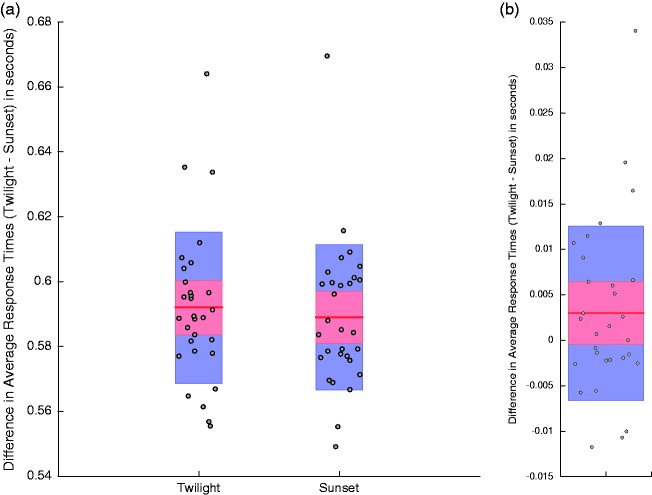
Details are as for Figure 2, but for data from blocks of trials completed while participants wore glasses with pink-tinted lenses.

**Figure 5.**
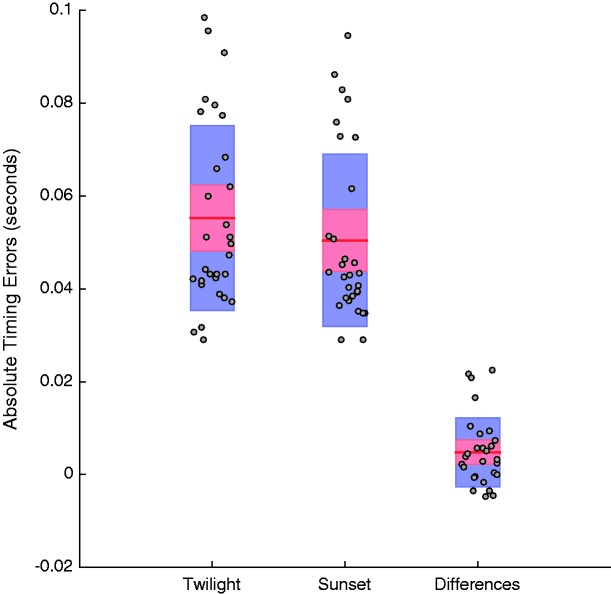
Average absolute timing errors for participants on trials in the Twilight and Sunset conditions, and differences between individual absolute timing errors. Individual data points are depicted by circular points, 95% CI limits by red shaded regions, and ± 1 *SD* by blue shaded regions. All data are from blocks of trials completed while wearing glasses with rose-tinted lenses.

Differences in average response times, on twilight (*M = *0.6 *SD* = 0.02; see Figure 4(a)) relative to sunset (*M = *0.6 *SD* = 0.02; see Figure 4(a)) trials, were eliminated when people wore glasses with rose-tinted lenses, *t*(29) = 1.7, *p* = .099, 95% CIs [−0.001, 0.007]; see Figure 4(b). People still, however, tended to make larger *absolute* timing errors on individual trials on twilight (*M* = 55 *SD* = 20 ms) relative to sunset (*M* = 50 *SD* = 19 ms) trials while they wore pink-tinted lenses, *t*(29) = 3.55, *p* = .0013, 95% CIs [0.002, 0.008]; see Figure 5. This means that while average response times were equated for these conditions, there was still greater trial-by-trial variance about the mean in the lower physical luminance contrast condition.

**Figure 6.**
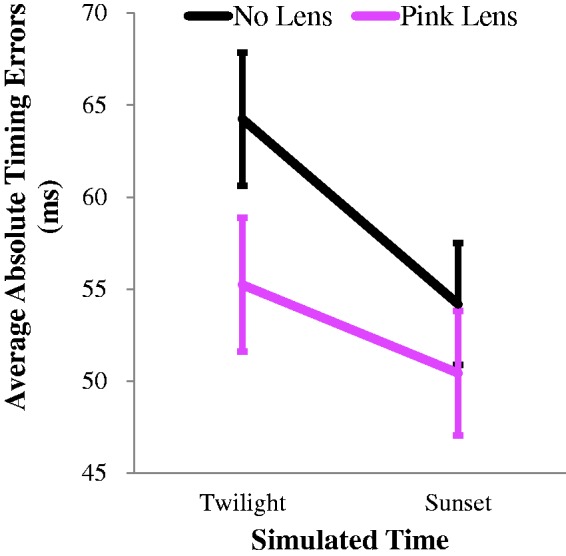
Mean absolute timing errors for participants in trials completed with and without pink-tinted lenses. Error bars depict ± 1 SEM.

To compare absolute timing errors across blocks of trials completed with and without pink-tinted lenses, we conducted a 2 × 2 repeated measures analysis of variance. This revealed a main effect for simulated time, *F*(1, 29) = 5.01, *p* = .032, η_p_^2 ^= .150, with greater absolute timing errors at twilight than at sunset (see Figure 6). There was also a main effect of tinted lenses, *F*(1, 29) = 34.47, *p* < .001, η_p_^2 ^= 0.543, with participants making smaller absolute timing errors while wearing pink-tinted lenses (see Figure 6).

## Discussion

Our data show that lighting conditions that mimicked the twilight period during a day/night cricket match (see Adie & Arnold, 2017) can adversely impact interceptive timing. People were slow to intercept a simulated pink cricket ball (see Figure 2) and made greater absolute timing errors (see Figure 3), both relative to a simulated sunset condition. The sunset condition was characterised by greater luminance contrasts between the pink ball and the pitch and the field relative to the twilight condition. Interceptive timing was, however, enhanced when people wore glasses with rose-tinted lenses. Wearing glasses eliminated differences in average interception times for the different lighting conditions (see Figure 4) and induced a general reduction in absolute timing errors (see Figure 6).

The implication of our data is that players and umpires might mistime cricket balls at twilight when there is little luminance contrast between balls and pertinent backgrounds (such as the field and pitch). This could put people at risk of injury, due to their inability to accurately judge ball speeds. In our simulations, these difficulties were mitigated by having people wear glasses with rose-tinted lenses. The precision of interceptive timing was enhanced in both the ‘twilight’ and ‘sunset’ lighting conditions (by ∼14% and 7%, respectively). We presume because the glasses relatively brightening the pink ‘ball’, thereby exaggerating luminance contrasts between the ball and equiluminant (and physically darker) backgrounds. Note, however, that this would be detrimental if the ball were darker than pertinent backgrounds – luminance contrasts would then be lessened by wearing the glasses.

The provision of trial-by-trial feedback probably minimised average interceptive timing differences between our lighting conditions (see Figure 2). We included feedback as we felt that this was akin to the real-world experiences of batsmen during play. While feedback might have mitigated timing differences across conditions, it did not eliminate them (see Figures 3 and 5). Average timing differences were, however, eliminated by artificially enhancing the luminance contrast of the pink ball relative to other coloured surfaces (see Figure 6). This implies that even when interceptive timing behaviour is (on average) accurate, precision can be enhanced by artificially exaggerating luminance contrasts, between the ball and different coloured backgrounds. This can be achieved easily in cricket by wearing glasses with appropriately tinted lenses. When the ball is darker than pertinent backgrounds lenses should relatively darken the ball. When the ball is brighter, lenses should further enhance this status. Our data suggest both situations could enhance the precision of interceptive timing.

While cricket motivated our experiments, our data have implications for other sports. Whenever a ball needs to be seen against a differently coloured background, chromatic filters could be used to exaggerate luminance contrasts and thereby enhance the precision of interceptive timing. In tennis, for instance, the precision of interceptive actions might be enhanced using lenses to exaggerate the luminance contrast of tennis balls relative to the court. This could either be achieved using glasses or contact lenses. In our experiments, interceptive timing precision was improved between 7% and 14% when using a chromatic lens. This would constitute a considerable advantage in professional sport.

It remains to be seen how well our data will generalise to real-world settings. In order to mimic luminance contrasts on our display device, we had to scale down physical luminance intensities relative to those recorded during a first-class cricket match (see Adie & Arnold, 2017). This can have a dramatic impact on motion processing (see Zele, Maynard, & Feigl, 2013). The dimensions and composition of retinal images in our task also differed from the perspective of a batsman in a real-world cricket match. Cricket balls must be tracked through three dimensions, from a moving hand, whereas discs in our experiments translated across one dimension, from a static point, across a two-dimensional display. We attempted to match physical luminance contrasts recorded during a real-world match, but did not attempt to match the viewpoint of a player. While noting these differences, we point out that motion perception is impacted by common computations in all contexts, and that motion in-depth is similarly adversely impacted by a lack of luminance contrast (see Cavanagh, Saidaj, & Rivest, 1995). We also only have light readings from a single cricket match. Lighting conditions would vary considerably, not only due to atmospheric conditions but also due to latitude, with the length and timing of twilight varying depending on where in the world a match is played. Our data do, however, capture a core anecdotal complaint of players and umpires – that the pink ball can be difficult to see at twilight. We would suggest it might be more accurate to say that the balls’ speed is more difficult to discern at twilight.

Obviously it would be desirable to conduct further research in this context. More light measurements should be taken in match conditions to develop a more accurate account of how conditions are impacted by playing through twilight and sunset. Further experiments should then be conducted, informed by a greater understanding of match conditions. These would clarify what impact lighting changes have on interceptive timing in cricket. We would normally hesitate, therefore, to advocate that participants take concrete actions on the basis of our preliminary findings. However, cricket is already being played with a pink ball during twilight, and players and umpires have raised anecdotal concerns about visibility (which accord with light readings we took during a match; see Adie & Arnold, 2017). These concerns are further supported by the interceptive timing data we have reported here. These matches can be regarded as a set of ongoing experiments, with players, umpires and the crowd as participants. We therefore suggest that if people experience difficulties seeing the pink ball about twilight, they might be well advised to try wearing ‘rose coloured’ glasses to artificially brighten the ball relative to pertinent backgrounds.
